# Slow angled-descent forepaw grasping (SLAG): an innate behavioral task for identification of individual experimental mice possessing functional vision

**DOI:** 10.1186/1744-9081-9-35

**Published:** 2013-08-23

**Authors:** Macarena Gil-Pagés, Robert J Stiles, Christopher A Parks, Steven C Neier, Maja Radulovic, Alfredo Oliveros, Alejandro Ferrer, Brendan K Reed, Katelynn M Wilton, Adam G Schrum

**Affiliations:** 1 Departamento de Psicología Biológica y de Salud, Programa de Licenciatura de Psicología, Universidad Autónoma de Madrid, Madrid, Spain; 2 Undergraduate Research Employment Program (UREP), Mayo Clinic, Rochester, MN, USA; 3 Initiative to Maximize Student Diversity (IMSD), Mayo Clinic, Rochester, MN, USA; 4 Summer Undergraduate Research Fellowship (SURF) program, Mayo Clinic, Rochester, MN, USA; 5 PhD program, Mayo Graduate School (MGS), Mayo Clinic, Rochester, MN, USA; 6 MD/PhD program, Mayo Medical School (MMS), Mayo Clinic, Rochester, MN, USA; 7 College of Medicine, Mayo Clinic, Rochester, MN, USA

**Keywords:** Vision, Innate behavior, SLAG, Dark chamber, C57BL/6, Mouse, Behavioral assay

## Abstract

**Background:**

There is significant interest in the generation of improved assays to clearly identify experimental mice possessing functional vision, a property that could qualify mice for inclusion in behavioral and neuroscience studies. Widely employed current methods rely on mouse responses to visual cues in assays of reflexes, depth perception, or cognitive memory. However, commonly assessed mouse reflexes can sometimes be ambiguous in their expression, while depth perception assays are sometimes confounded by variation in anxiety responses and exploratory conduct. Furthermore, in situations where experimental groups vary in their cognitive memory capacity, memory assays may not be ideal for assessing differences in vision.

**Results:**

We have optimized a non-invasive behavioral assay that relies on an untrained, innate response to identify individual experimental mice possessing functional vision: slow angled-descent forepaw grasping (SLAG). First, we verified that SLAG performance depends on vision and not olfaction. Next, all members of an age-ranged cohort of 158 C57BL/6 mice (57 wild-type, 101 knockout, age range 44–241 days) were assessed for functional vision using the SLAG test without training or conditioning. Subjecting the population to a second innate behavioral test, Dark Chamber preference, corroborated that the functional vision assessment of SLAG was valid.

**Conclusions:**

We propose that the SLAG assay is immediately useful to quickly and clearly identify experimental mice possessing functional vision. SLAG is based on a behavioral readout with a significant innate component with no requirement for training. This will facilitate the selection of mice of known sighted status in vision-dependent experiments that focus on other types of behavior, neuroscience, and/or cognitive memory.

## Background

The visual system is of outstanding interest in behavioral and neural sciences. Historically, the anatomy of the eye and its neuronal associations made the system accessible to mapping the pathways that encode sensation and perception of an external stimulus in the brain [1-3]. Modern experimentation in behavioral neuroscience often relies on test subjects’ visual capacity to accomplish requisite tasks. In mice, as nocturnal rodents, olfaction and hearing are considered the more dominant senses for perception of objects at a distance; however, mouse vision is appreciated as an important contributing sense despite an estimated 20/2000 acuity [4,5].

Cognitive memory experiments in mice often require the measurement of responses to visual cues. Because of this, such memory assays themselves can sometimes be used to detect differences in mouse visual acuity [6-8]. This requires the assumption or demonstration that the mice involved possess equivalent memory capacity, allowing differences in performance to be attributed to differences in vision. Common memory assays that have been applied in this way include some that are maze-based, or involve Pavlovian cue/context fear conditioning, or conditioned suppression of specific behaviors [9]. However, quite often the converse experiment is desirable, such that cognitive memory capacity can be treated as the variable, when baseline visual performance can be considered equivalent between responding mice.

There is a recognized need in the field for assays that would improve identification of individual mice possessing functional vision that are to be used in subsequent behavioral/memory experiments [9]. Ideally, such assays should be robust, reproducible, simple, and economical, require no mouse behavioral training or conditioning, and require no behavior-altering procedures such as whisker (vibrissae) trimming or tail amputation. Two common procedures examine vision-based behavioral reflexes in the mouse: eye-blink and visual placing (forepaw-reaching) tests [10]. Both invoke a response to an object approaching the eye: in the eye-blink test, a cotton swab approaching the eye induces the mouse to wince or blink, while in the visual placing test, descent of a suspended mouse toward an incoming flat surface induces a forward stretching motion of the forepaws. However, vision is not the only sense that can induce these responses, which can also occur if the whiskers or nose are touched during either procedure. Furthermore, expression of these reflexes in sighted mice can sometimes appear ambiguous. A third reflex-based functional vision assessment tool has not yet been adopted for general use as a pre-test in cognitive behavioral studies, but holds outstanding promise for potential general application in this field: Optokinetic tracking involves assessment of the optokinetic reflex with an optokinetic drum [11-15]. Conceivably, the instrumentation and procedures involved could be optimized and/or validated in a minimally invasive, behavior non-modifying format to identify individual sighted mice for subsequent behavioral experimentation.

Beyond reflexes, two common tests rely on untrained behavioral responses to visual depth perception: visual cliff [16] and elevated-plus maze [17] tests. However, since up to 10% of mice from the best performing strains can fail, these tests may be most suitable for general strain characterization, while they somewhat more cautiously supply the sighted vs. blind status for each individual mouse [9]. Vision scoring errors on these tests may occur due to the use of other senses to perceive and judge distances, or variation in innate fear vs. exploratory impulses during task performance [18].

We have prepared a simple, economical, behavioral assay that uses an untrained, innate behavioral response to identify individual experimental mice that possess functional vision: slow angled-descent forepaw grasping (SLAG). At the population level, SLAG vision assessment can be validated by a second assay that is based on a different innate response, Dark Chamber preference. Furthermore, we show that SLAG is compatible with the C57BL/6 (B6) background, the most common strain used in neuroscience experiments involving genetic engineering. It is anticipated that this assay will facilitate the selection of individual mice of known sighted status, which can be subsequently destined for experiments focusing on other behavioral and cognitive variables.

## Results and discussion

### Slow angled-descent forepaw grasping (SLAG)

**Figure 1:**
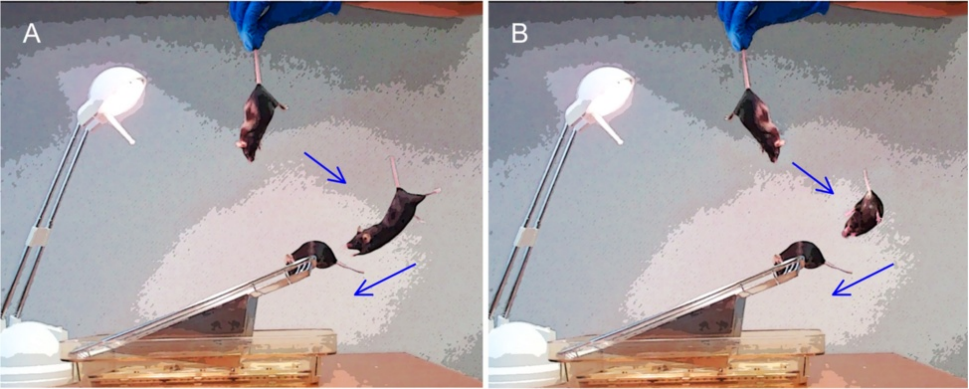
**Slow angled-descent forepaw grasping (SLAG). (A)** Approximately 15–30 cm above an illuminated wire-bar cage lid, the mouse is suspended by the tail with its ventral aspect and the raised edge of the lid toward the same side (from the viewer’s perspective, the right side). The mouse is slowly lowered according to the angle depicted by the blue arrows, and in this orientation, the wire lid remains within the mouse’s field of view throughout descent. Upon nearly passing the edge of the wire lid, the SLAG(+) mouse will stretch forth its forepaws in a sustained fashion toward the wire, whereas a SLAG(−) mouse (not depicted) will not. Finally, regardless of its performance, the mouse is placed on the wire lid for several seconds. **(B)** The test is immediately repeated, but with the mouse’s dorsal aspect toward the same side as the raised edge of the lid (from the viewer’s perspective, the right side). In this orientation, the wire lid becomes excluded from the mouse’s field of view when the descending mouse begins to pass the raised edge; the SLAG(+) mouse will twist around to extend its forepaws toward the wire lid in a sustained fashion, whereas a SLAG(−) mouse (not depicted) will not.

**Figure 2:**
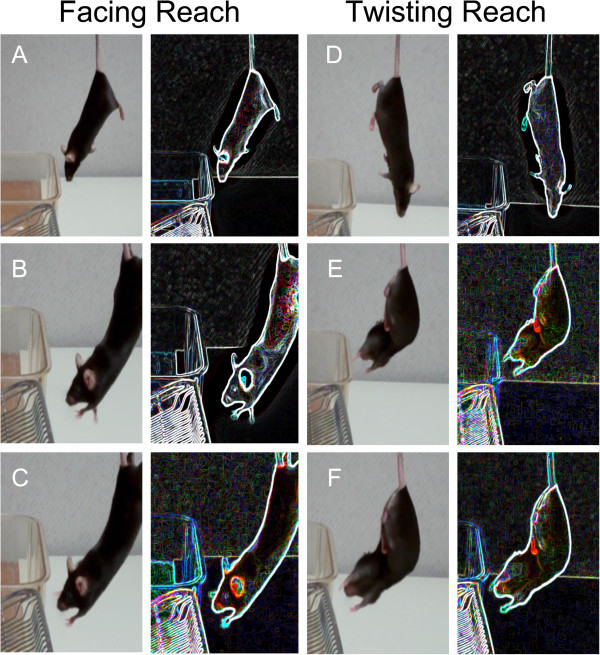
**SLAG(+) task performance.** Original still shots (left) from Additional file 1 are accompanied by modifications using the “Glowing Edges” stylization function of Adobe Photoshop software (right), to enhance visualization of the mouse’s paws in relation to the wire lid. During the first descent, the mouse does not reach toward the wire lid **(A)** until it begins to become close **(B)**, leading to obvious, sustained forepaw extension **(C)**. Finally, the mouse is permitted to grasp the wire lid and remain on it for several seconds. The task is repeated with the mouse in opposite horizontal orientation. Upon the second descent, the mouse does not immediately reach toward the wire lid **(D)**, but as it gets closer, the mouse twists its body around to reach **(E)**, until an obvious, sustained, directed manner is observed **(F)**. Finally, the mouse is permitted to grasp the wire lid as the test is completed.

SLAG was performed in the following manner (Figure 1): A low-heat work lamp was positioned to illuminate a clean, wire-bar stainless steel cage lid, one edge of which had been set at an upward angle. Each mouse was suspended by the tail approximately 15–30 cm above the lid, such that the mouse’s ventral aspect and the raised edge of the lid were toward the same side (from the viewer’s perspective, the right side in Figure 1A). In this orientation, the mouse was slowly lowered vertically while passing over the wire lid horizontally, resulting in a diagonal descent, while the wire lid remained within the mouse’s field of view throughout. Eventually, the mouse passed over the raised edge of the wire lid within ~4 cm, and clearly outside the distance where its whiskers might touch the lid. At this point, SLAG(+) mice displayed a behavior that involved directed, sustained reaching of the forepaws toward the raised edge of the wire lid, while SLAG(−) mice did not (Figure 2A-C, Additional file 1). Regardless of which behavior was observed, each test mouse was then placed briefly on the wire lid. Next, the test was repeated, however the horizontal orientation of the mouse was reversed, such that the mouse’s dorsal aspect and the raised edge of the lid were toward the same side (from the viewer’s perspective, the right side in Figure 1B). This caused the wire lid to become excluded from the mouse’s field of view as its trajectory upon descent began to pass the raised edge. SLAG(+) mice displayed a behavior that involved twisting around to extend the forepaws toward the wire cage in a sustained and directed manner, while SLAG(−) mice did not (Figure 2D-F, Additional file 1).

The SLAG scoring outcome usually appeared obvious, but it involved subjective judgment by the observer. Therefore, we wished to perform an inter-rater reliability test to examine the scoring variability when multiple observers witnessed identical SLAG sessions. First, a series of B6 mice were pre-screened to obtain their SLAG score, (+) or (−), and a cohort was chosen that insured inclusion of both kinds of mice. Next, SLAG was repeated for this cohort with the assay sessions recorded on video. Nine volunteers were educated in the SLAG procedure and scoring, by (i) studying an early version of this manuscript, (ii) witnessing a demonstration session with an expert, and (iii) practicing on live mice to become familiar with the technique. Two of the participants had a high level of SLAG expertise, while the others did not. All nine participants independently viewed the same collection of 22 videos and scored the mice. We found that all raters produced the same SLAG scores in every case except one (Table 1), with Light’s modified Cohen κ-statistic for inter-rater reliability [19] equal to 0.98 (where 1.0 is perfect agreement and −1.0 is perfect disagreement). We conclude that although the scoring method is subjective, activity in the SLAG assay is perceived with little variation by multiple observers.

**Table 1:** Inter-rater reliability with multiple observers witnessing identical SLAG sessions

	**Rater**								
**Mouse**	**A**	**B**	**C**	**D**	**E**	**F**	**G**	**H**	**I**
1	**+**	**+**	**+**	**+**	**+**	**+**	**+**	**+**	**+**
2	**-**	**-**	**-**	**-**	**-**	**-**	**-**	**-**	**-**
3	**+**	**+**	**+**	**+**	**+**	**+**	**+**	**+**	**+**
4	**+**	**+**	**+**	**+**	**+**	**+**	**+**	**+**	**+**
5	**-**	**-**	**-**	**-**	**-**	**-**	**-**	**-**	**-**
6	**+**	**+**	**+**	**+**	**+**	**+**	**+**	**+**	**+**
7	**+**	**+**	**+**	**+**	**+**	**+**	**+**	**+**	**+**
8	**-**	**-**	**-**	**-**	**-**	**-**	**-**	**-**	**-**
9	**-**	**-**	**-**	**-**	**-**	**-**	**-**	**-**	**-**
10	**-**	**-**	**-**	**-**	**-**	**-**	**-**	**-**	**-**
11	**+**	**+**	**+**	**+**	**+**	**+**	**+**	**+**	**+**
12	**+**	**+**	**+**	**+**	**+**	**+**	**+**	**+**	**+**
13	**-**	**-**	**-**	**-**	**-**	**-**	**-**	**-**	**+**
14	**+**	**+**	**+**	**+**	**+**	**+**	**+**	**+**	**+**
15	**+**	**+**	**+**	**+**	**+**	**+**	**+**	**+**	**+**
16	**-**	**-**	**-**	**-**	**-**	**-**	**-**	**-**	**-**
17	**+**	**+**	**+**	**+**	**+**	**+**	**+**	**+**	**+**
18	**+**	**+**	**+**	**+**	**+**	**+**	**+**	**+**	**+**
19	**-**	**-**	**-**	**-**	**-**	**-**	**-**	**-**	**-**
20	**-**	**-**	**-**	**-**	**-**	**-**	**-**	**-**	**-**
21	**+**	**+**	**+**	**+**	**+**	**+**	**+**	**+**	**+**
22	**+**	**+**	**+**	**+**	**+**	**+**	**+**	**+**	**+**

Nine people (raters A-I, columns) were familiarized with SLAG performance and scoring techniques. Each participant independently viewed a collection of videos of SLAG being performed on 22 mice (rows), and scored each mouse as (+) or (−). All scores except for one coincided between raters. Light’s modified Cohen κ-statistic for inter-rater reliability [19] was calculated to be 0.98, indicating a high level of scoring agreement between raters.

### SLAG(+) performance depends on vision

**Figure 3:**
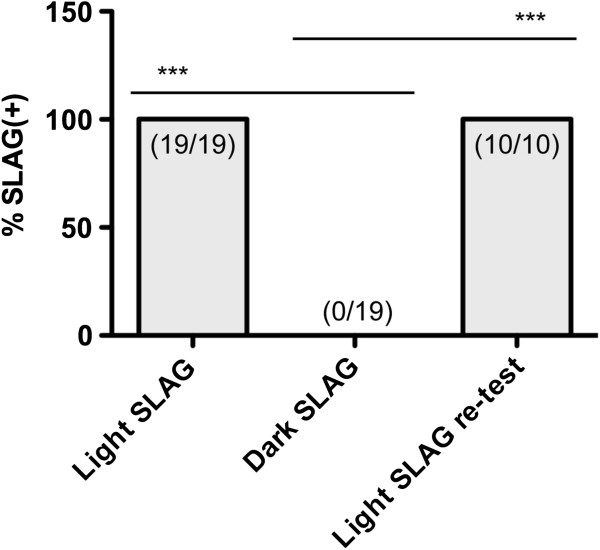
**SLAG(+) performance requires ambient light.** 19 B6 mice were pre-identified as SLAG(+), and this scoring was confirmed upon repeating the test under normal conditions that include ambient light (Light SLAG). Upon removing ambient light, no mouse produced SLAG(+) behavior, as observed by an experimenter using infrared night vision goggles (Dark SLAG). Finally, ambient light was returned, and of 10 mice re-tested, all reproduced SLAG(+) behavior (Light SLAG re-test). One-tailed Chi-square analysis showed statistical differences between Light SLAG and Dark SLAG groups: *** p < 0.0001.

We wished to determine whether vision was involved in sensing the presence of the wire lid during SLAG(+) performance. Nineteen B6 mice that had already been scored as SLAG(+) were tested again, and all mice repeated the SLAG(+) behavior (Figure 3). Next, the test was performed on the same mice in the dark, while the experimenter observed using infrared night vision goggles. We found that it was possible for a mouse to retain SLAG(+) behavior under these conditions, if some ambient light remained in the procedure room, originating from an adjacent hallway and passing through the junctions between door and wall (data not shown). This demonstrated that the presence of the night vision goggles and their use by the experimenter did not inhibit SLAG(+) behavior *per se*, nor prevent the experimenter from observing the behavior. However, when all ambient light was removed, by applying duct tape around the door junctions, all mice failed to demonstrate SLAG(+) behavior (Figure 3). Next, lighting was returned to the room to re-test 10 mice, and all 10 re-demonstrated SLAG(+) behavior (Figure 3). We conclude that SLAG(+) performance is dependent on ambient light, and thus requires vision.

**Figure 4:**
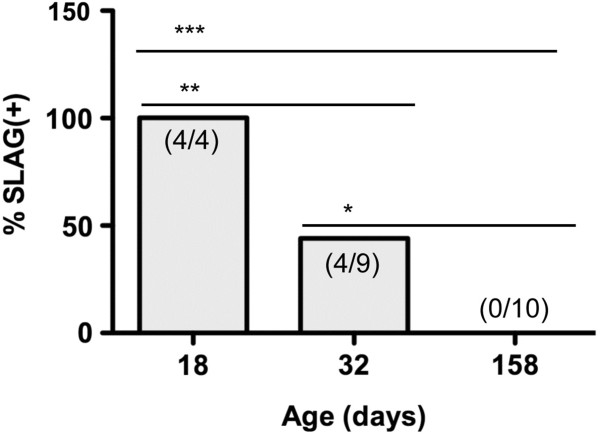
**FVB mice are SLAG(+) when young, but become SLAG(−) upon aging.** Female FVB mice of three different ages (x-axis) were subjected to SLAG testing. Each bar graph shows the number of SLAG(+) / total number of mice: 4/4, age 18 days; 4/9, age 32 days; 0/10 age 158 days. The bar graphs display the percentage of SLAG(+) mice for each age group. One-tailed Chi-square analysis showed statistical differences between age groups: *** p < 0.0001, ** p = 0.008, * p = 0.03.

If correct, this predicted that mice that were blind due to genetic mutation would score as SLAG(−). To test this hypothesis, we used wild-type FVB mice, which are known to bear a retroviral insertion and nonsense mutation interrupting the *Pde6b*^rd1^ locus [20]. While young FVB mice are sighted, retinal degeneration, in concert with other albinism-associated genes, causes their vision to erode substantially by one month of age [21]. We observed that although young FVB mice (18 days of age) scored as SLAG(+), approximately half (44%) of older mice (32 days of age) tested in parallel were SLAG(−), and all of the oldest mice (158 days of age) tested in parallel were SLAG(−) (Figure 4). These observations correlate loss of eyesight with loss of SLAG(+) performance, consistent with the idea that SLAG(+) performance requires functional vision.

**Figure 5:**
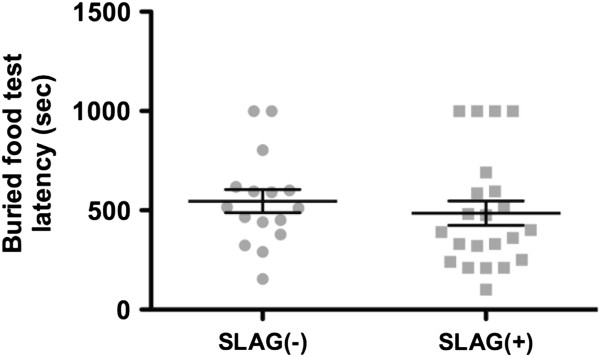
**SLAG(+) performance does not correlate with differences in olfaction.** A cohort of 38 B6 mice was subjected to both SLAG and a buried-food olfactory test. All mice in the cohort included 16 SLAG(−) mice and 22 SLAG(+) mice. The mean latency to find buried food was 546 sec for SLAG(−) and 486 sec for SLAG(+) mice, which was not significantly different (unpaired two-tailed t-test, p = 0.49; mean +/− SE bars displayed).

In contrast, we found that SLAG(+) behavior did not correlate with potential differences in olfaction capability. A cohort of 38 B6 mice was subjected to SLAG, and subsequently a buried-food olfactory test, measuring the time required for a fasting mouse to locate food hidden underneath cage bedding [22]. We found that there was no statistically significant difference in buried-food performance between SLAG(+) and SLAG(−) mice (Figure 5). We propose that vision, not olfaction, is the primary sense involved in SLAG performance.

### SLAG identifies individual mice possessing functional vision, as validated by the dark chamber (DC) preference test

We wished to determine how the SLAG test might correlate with a second innate behavioral response that assesses mouse vision at the population level. To do this, we set up a light/dark chamber apparatus, where one chamber was illuminated, the other dark, with the two chambers separated by a plastic wall and an opening sufficient for a mouse to pass freely between them [23]. In a ten minute time period, mice are expected to display exploratory behavior in both chambers, but sighted mice would spend more time in the dark chamber, averse to the light, while blind mice would spend equal time in both chambers. The B6 strain in particular has previously shown significant exploratory behavior in both light and dark chambers [24]. A limitation of the DC preference test is that although it can reveal visual deficiencies, it does not distinguish whether such deficiencies may be in image-forming vs. non-image-forming photoreception. The DC test depends on behavioral aversion to light, and this response depends more on melanopsin-positive intrinsically photosensitive retinoganglion cells than on the retinal rod and cone photoreceptors that mediate image-forming vision [25-27]. Never the less, it has been reported that mice with deficiencies in either image-forming or non-image-forming vision will fail the DC test [28], revealing at least one type of deficiency, and thus we considered the DC test to be adequate as a validation assessment for SLAG data.

To consider SLAG in conjunction with DC preference, we assessed an age-ranged cohort of 158 B6 mice (57 wild-type, 101 knockout, age range 44–241 days). Knockout mutations were focused on subunits of the Major Histocompatibility Complex (MHC) class I (β2m^−/−^ or H2KbDb^−/−^), subunits of the CD3 complex (δ^−/−^ or ϵ^−/−^ζ^−/−^), and Recombination Activating Gene 2 (RAG2^−/−^). Although often studied in the context of the adaptive immune system, MHC class I and CD3 knockout mice have been previously shown to display defects in the synaptic pruning that occurs during developmental remodeling of retinal afferent projections [29-32]. Separate studies show that MHC class I knockout mice also possess alterations in motor learning capacity [33]. In contrast, RAG^−/−^ mice do not display either the retinal development or learning alterations despite their lack of adaptive immune lymphocytes. These previously published observations argue that MHC and CD3 possess functional roles intrinsic to the nervous system that are not sequelae of their immune functions. Because the present mice were not reared under conditions of light deprivation, it does not necessarily follow that their mutations should produce blindness [34]. Never the less, it has not been previously shown whether adult MHC class I and CD3 knockout mice are blind. For the present study, our goal was to clearly identify sighted mice, to allow the inclusion of individuals of known vision function in subsequent cognitive memory/behavioral experiments.

**Figure 6:**
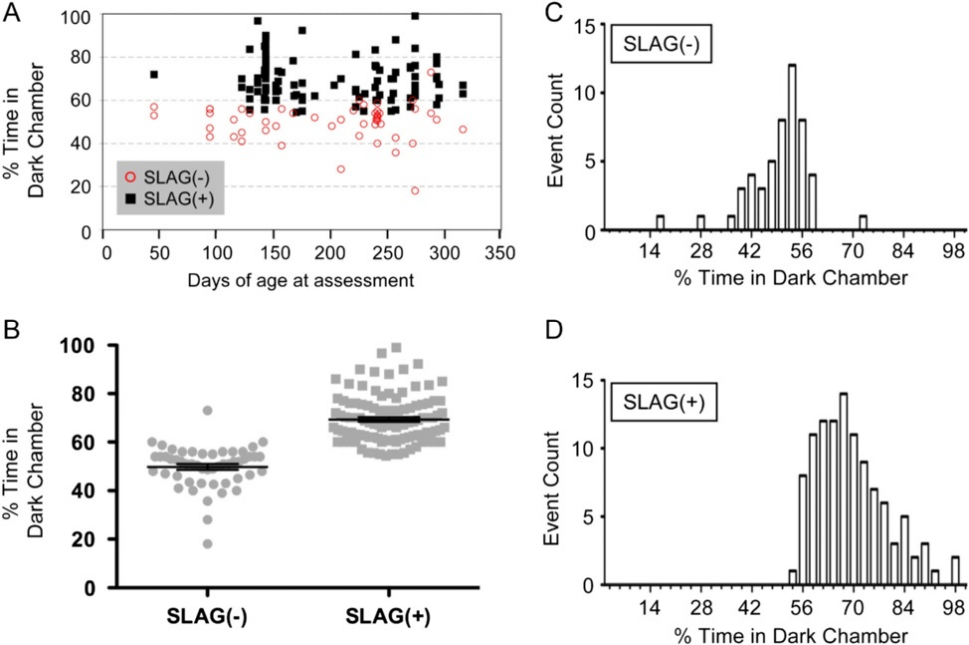
**SLAG identifies individual mice possessing functional vision, as validated by DC preference test.** Data are pooled together from an age-ranged cohort of 158 B6 mice (57 wild-type, 101 knockout, age range 44–241 days; separate data for individual mice from each genotype appear in Figure 7). **(A)** Visualization of SLAG and DC performance as a function of age. No evidence for correlation between age and DC preference was observed: SLAG(−) R^2^ = 0.0012, SLAG(+) R^2^ = 0.0135 (regression lines not shown). **(B)** Mean time spent in DC: SLAG(−) = 50%, SLAG(+) = 69% (unpaired two-tailed t-test, means are significantly different, p < 0.0001; two-tailed Mann–Whitney test, p < 0.0001; mean +/− SE bars displayed). Note that every SLAG(+) mouse spent >50% time in DC. **(C)** Histogram visualization of the data in (B) shows that the SLAG(−) population appears roughly symmetrical, passing the Kolmogorov-Smirnov (KS) test for normality (α = 0.05). **(D)** Histogram visualization of the data in (B) shows that the SLAG(+) population follows an asymmetrical distribution with respect to DC preference, failing the KS test for normality (α = 0.05).

**Figure 7:**
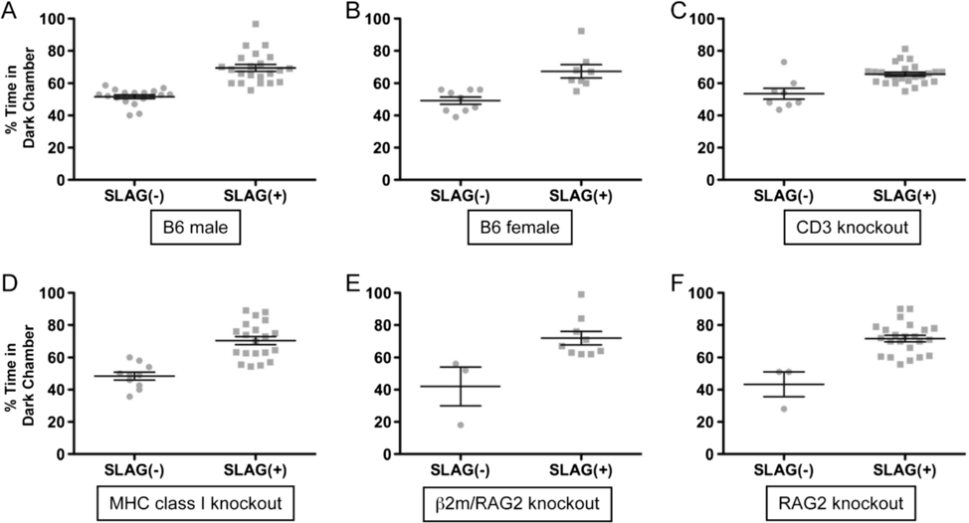
**SLAG performance correlates with Dark Chamber (DC) preference across various experimental groups.** All experimental groups compared here displayed a statistically significant increase in DC preference for SLAG(+) compared with SLAG(−) mice (means +/− SE bars displayed). **(A)** For B6 males, mean time spent in DC: SLAG(−) = 52%, SLAG(+) = 69% (unpaired two-tailed t-test, means are significantly different, p < 0.0001; two-tailed Mann–Whitney test, p < 0.0001). **(B)** For B6 females, mean time spent in DC: SLAG(−) = 49%, SLAG(+) = 67% (unpaired two-tailed t-test, means are significantly different, p = 0.0011; two-tailed Mann–Whitney test, p = 0.0017). **(C)** CD3 knockout mice include CD3 δ^−/−^ and CD3ϵ^−/−^ζ^−/−^ pooled together. Mean time spent in DC: SLAG(−) = 54%, SLAG(+) = 66% (unpaired two-tailed t-test, means are significantly different, p = 0.0002; two-tailed Mann–Whitney test, p = 0.0019). **(D)** MHC class I knockout mice include β2m^−/−^ and H-2KbDb^−/−^ pooled together. Mean time spent in DC: SLAG(−) = 48%, SLAG(+) = 70% (unpaired two-tailed t-test, means are significantly different, p < 0.0001; two-tailed Mann–Whitney test, p < 0.0001). **(E)** For β2m/RAG2 double knockout mice, mean time spent in DC: SLAG(−) = 42%, SLAG(+) = 72% (unpaired two-tailed t-test, means are significantly different, p = 0.0116; two-tailed Mann–Whitney test, p = 0.016). **(F)** For RAG2 knockout mice, mean time spent in DC: SLAG(−) = 43%, SLAG(+) = 72% (unpaired two-tailed t-test, means are significantly different, p = 0.0001; two-tailed Mann–Whitney test, p = 0.0063).

We found that SLAG and DC performance correlated as predicted if both tests assess vision, with SLAG(+) mice spending significantly more time in the dark chamber than SLAG(−) mice (Figure 6A-D). This was true for wild-type B6 males (Figure 7A) and females (Figure 7B), and mice knocked out for CD3 (Figure 7C), MHC class I (Figure 7D), MHC class I (β2m) and RAG2 (Figure 7E), or RAG2 alone (Figure 7F). Pooling all data together showed that both SLAG(−) and SLAG(+) mice were distributed across the age range as two distinct populations for DC performance (Figure 6A). There was no tendency for decrease in SLAG(+) or DC preference with increasing age, either in the pooled population (low R^2^ values, Figure 6A legend) or in separate experimental groups (data not shown). Viewing the pooled data without age stratification (Figure 6B), we observed that SLAG(−) mice spent an average of 50% time in the dark chamber, precisely as predicted for blind mice. SLAG(+) mice spent an average of 69% time in the dark chamber, preferring it over the lighted chamber as predicted for sighted mice. Furthermore, every SLAG(+) mouse spent >50% time in DC. Upon examination of the pooled data in histogram format, we observed that SLAG(−) time in DC produced a distribution with apparent symmetry, passing the Kolmogorov-Smirnov (KS) test for normality (Figure 6C). In contrast the SLAG(+) time in DC produced an asymmetric distribution, failing the KS test for normality (Figure 6D). These properties describe two statistically distinct populations. We conclude that the population analysis of the DC preference test validated the individual mouse analysis of the SLAG test, resulting in clear identification of sighted mice.

### Concluding remarks

The SLAG test appears capable of identifying individual mice possessing functional vision. Considering SLAG together with DC preference, both tests depend on light perception, but the responses to these tests do not represent reflexes. Because neither test requires mouse training or conditioning, the easily legible behavioral responses likely contain a significant innate component. We propose that the SLAG test is immediately useful for identifying sighted mice, and using this information to qualify mice for further testing of other behavioral and cognitive variables such as memory. It remains possible that positive performance on the SLAG test may not indicate precise visual equivalence between mice destined for tests that may involve other visual stimuli, such as those used in Morris water maze or standard operant chambers. However, when studying mutations that may affect both the visual system and cognitive memory (such as MHC class I or CD3), we propose that mice of equivalent SLAG performance can be included in memory experiments, for increased accuracy in attributing the outcome of such testing to differences in memory itself.

## Methods

### Mice

Wild-type and knockout mice were on the C56BL/6 (B6) strain background, except for wild-type FVB mice (Figure 4). Wild-type B6 mice were either purchased from the Jackson Laboratory, or were the offspring of breeder colonies from founders purchased within the last three years. Wild-type FVB mice were bred and aged in our facility, originating from the colony of Chella David (Mayo Clinic), who has bred the colony continuously for 19 years since receiving FVB/NJ founders from the Jackson Laboratory. CD3δ knockouts were bred in our facility, originally provided by Dietmar Kappes (Fox-Chase Cancer Center). CD3ϵζ knockouts were bred in our facility, originally provided by Dario and Kate Vignali (St. Jude Children’s Hospital) with permission from Cox Terhorst (Beth Israel Deaconess Medical Center). H-2KbDb knockouts were bred in our facility, originally provided by Larry Pease (Mayo Clinic). β2m and RAG2 knockouts were bred in our facility, and β2m/RAG2 double knockouts were bred as a colony originating from single knockout progenitors. The first cohort of B6 mice (Figure 3) included 17 males aged 60–70 days, and 2 males aged 215–225 days. The large cohort (all other data) comprised 158 B6 mice including both males and females, age range 44–241 days (57 wild-type, 13 CD3δ^−/−^, 20 CD3ϵ^−/−^/ζ^−/−^, 20 H-2KbDb^−/−^, 10 β2m^−/−^, 12 β2m^−/−^/RAG2^−/−^, 26 RAG2^−/−^). The buried-food olfactory test (Figure 5) used a subset of 38 wild-type mice from the large cohort. All animal procedures were performed in accordance with IACUC regulations at Mayo Clinic.

### Mouse general health, acclimatization, and inclusion in the study

Mice were housed in Mayo’s specific pathogen-free facility. Because some mice in the large cohort were genetically immune-compromised, beginning one month before and throughout the period of experimentation, all mice in that cohort were fed Teklad Global 2018 (Uniprim 4100 ppm) medicated, irradiated chow, containing Trimethoprim (275 ppm) and Sulfadiazine (1365 ppm) (Harlan). All mice were provided clean cages with fresh food and water on one fixed day per week, and no experimentation was performed on that day. Mice were assessed for general appearance (grooming, bald spots, missing whiskers, and coat appearance) and general health (weight, whiskers reflex, righting reflex, acoustic startle reflex). These assessments were made in several preliminary sessions, during which mice were acclimated to the transportation routine between general housing and the procedure room, and to experimenter handling. The transportation routine included waiting one hour after transport to the procedure room before commencing handling and experiments. After any session with an experimenter, a generic-brand sweetened cereal piece (Tootie Fruities, Wal-Mart Stores, Inc.) was placed in the home cage prior to return to the general housing room. This habituated the mice to the cereal, which was later used in the Buried Food Olfactory Test (described below). Mice that appeared healthy were included in the studies.

### Slow angled-descent forepaw grasping (SLAG)

Details of this procedure are found in the general text. The low-heat (20W, 12V) Espressivo Work Lamp (IKEA) was used for illumination. The clean, wire-bar stainless steel cage lids (Allentown Inc.) were taken from the same pool used for routine housing, to insure that the mice would be habituated to the object. Between mice, either the wire lid was wiped with 70% ethanol and water, or replaced with a fresh lid. For Dark SLAG, infrared night vision goggles (Viper model, ATN) were used with a headmount so that the experimenter’s hands were free to perform the procedure during observation.

### Dark chamber (DC) preference test

The concept that sighted mice display a preference for a dark chamber over a lighted chamber is well known [23]. A rat cage divider was constructed from cut corrugated plastic, 2.5 mm thick, snugly fitting the transverse dimensions of the cage hollow, with a 7×7 cm square opening at the bottom center. The divider was placed in the middle of a standard rat cage (259 mm × 476 mm × 209 mm, PC10198HT, Allentown Inc.), separating the cage into two equally sized chambers. One chamber was covered along the outside with black plastic that was secured with black tape, while the other chamber had the same tape applied on its outside surface in an equal pattern. The low-heat lamp was positioned above the cage divider, so that it illuminated both chambers equally on the outside, while the black plastic blocked light from entering the inside of its associated chamber. A filterless, coarse mesh cage top was placed over the cage, and under these conditions, the luminosity in each chamber was 21.5 lux (dark chamber) and 237 lux (lighted chamber). For experiments, a mouse was released in the lighted chamber and was free to pass between lighted and dark chambers *ad libitum*. The duration of the test was 10 minutes, during which the time spent in the lighted chamber was recorded with a stopwatch by the experimenter. The apparatus was cleaned with 70% ethanol followed by water between all test subjects.

### Buried-food olfactory test

This was performed following published methods [22] with slight modification. Mice were housed 24h without food prior to the test, which was performed in a rat cage with 2.5 cm bedding and a coarse mesh top. A piece of generic-brand sweetened cereal (to which the mice were already habituated) was buried in the bedding at a single, randomly selected position used in all experiments. The experimenter measured the time elapsed (latency) to find the food, with a maximum test duration of 999 seconds. The test cage was cleaned with 70% ethanol followed by water, and new bedding was provided between all test subjects.

### Video and photos

A Flip Ultra-HD digital video camera (Cisco Systems) was used to record video. The original video footage was edited to compose Additional file 1 using Wondershare™ freeware. Still shots from the original video footage were captured using the screenshot function of Macintosh OS 10.6.8 desktop software, and were used to compose Figure 2. Adobe Photoshop software was used for the following: (a) to prepare the original still shots for figures; (b) to prepare the demonstrative cartoon illustration of SLAG (Figure 1); (c) to apply the “glowing edges” stylization for enhanced visualization of the positioning of the mouse paws with respect to the wire lid during the SLAG procedure (Figure 2).

### Statistics

Student’s t-test, Chi-square, Mann–Whitney, Kolmogorov-Smirnov (KS) test for normality, and Regression (R^2^) analyses were performed using Prism Graphpad and Microsoft Excel software. Light’s modified Cohen κ-statistic for inter-rater reliability was calculated as described [19].

## Abbreviations

B6: C57BL/6 mouse strain; CD3: Cluster of differentiation 3 (“CD” is a nomenclature used for cell-expressed proteins with relevance to the immune system.); DC: Dark Chamber preference test; KS: Kolmogorov-Smirnov test for normality; MHC: Major histocompatibility complex; RAG: Recombination activating gene; SLAG: Slow angled-descent forepaw grasping.

## Competing interests

The authors declare no competing interests.

## Authors’ contributions

MGP conceived, designed and performed experiments, analyzed data, and wrote the manuscript. RJS designed and performed experiments, and analyzed data. CAP performed experiments and provided essential design elements used in mouse habituation to the procedure room, routine, and experimenters. SCN designed and performed experiments, and analyzed data. MR, AO, AF, BKR, and KMW performed experiments. AGS conceived and designed experiments, analyzed data, and wrote the manuscript. All authors read and approved the final manuscript.

## Supplementary Material

Additional file 1 **Supplemental Video 1.** Demonstration of the slow angled-descent forepaw grasping (SLAG) test for identification of sighted mice. The left side of the screen shows a SLAG(+) mouse, while the right side shows a SLAG(−) mouse. Approximately 15–30 cm above an illuminated wire-bar cage lid, each mouse is suspended by the tail with its ventral aspect oriented toward the same side as the raised edge of the lid (from the viewer’s perspective, the right side). The mouse is slowly lowered over and past the wire lid, and in this orientation, the wire lid remains within the mouse’s field of view during descent. Upon nearly passing the edge of the wire lid, the SLAG(+) mouse (left) stretches forth its forepaws in a sustained fashion toward the wire lid, whereas the SLAG(−) mouse (right) does not. Regardless of its performance, each mouse is briefly placed on the wire lid (not shown for the SLAG(−) mouse on the right). The test is immediately repeated, but with the mouse starting in the opposite horizontal orientation. Upon descent, the wire lid becomes excluded from the mouse’s field of view when the mouse passes the raised edge, and the SLAG(+) mouse (left) twists around to extend its forepaws toward the wire lid in a sustained fashion, whereas the SLAG(−) mouse (right) does not. The video bears the watermark of Wondershare™, the freeware used to convert the video into a format compatible with the memory and accessibility requirements of the journal. Click here for file
